# Investigation on Patient/Compensator Scatter Factor for Monitor Unit Calculation in Proton Therapy

**DOI:** 10.14338/IJPT-18-00021.1

**Published:** 2018-11-30

**Authors:** Michael T. Prusator, Salahuddin Ahmad, Yong Chen

**Affiliations:** Department of Radiation Oncology, University of Oklahoma Health Sciences Center, Oklahoma City, OK, USA

**Keywords:** monitor unit, Monte Carlo, proton, TOPAS

## Abstract

**Purpose:**

It is the goal of this study to use both Monte Carlo (MC) simulation and the pencil beam dose algorithm (PBA) in the treatment planning system to investigate Patient scatter factor (PSF) and Compensator scatter factor (CSF) for calibrating the dose per monitor unit (DMU) for a passive scattering proton therapy system.

**Materials and Methods:**

PSFs and CSFs for brain, lung, pancreas, and prostate treatment sites were calculated by using MC simulation and PBA from the treatment planning software to evaluate the agreement between the two.

**Results:**

This study shows that the CSF values are always greater than 1, with some reaching nearly 4% above unity, and depending strongly on the shape of the compensator. Monte Carlo and PBA-calculated CSF factors agree very well, with average differences below 1%. PSF values calculated in this study ranged from 0.919 to 1.023 and are largely dependent on the type of tissue heterogeneities in the treatment field. Monte Carlo and PBA-calculated PSF factors show differences, with the largest discrepancies seen in lung cases, with an average difference of 1.9%. It is also shown that dense bone will drive a PSF to values greater than unity, while large quantities of air decrease the PSF to below unity.

**Conclusion:**

We have showed that the compensator and patient anatomy can have a significant impact on clinical proton dose distribution. It is recommended that both Monte Carlo and treatment planning system should be used to take these factors into account in the final DMU calculation.

## Introduction

Proton therapy is becoming an increasingly popular method for the treatment of cancer. The sharp dose characteristics of the Bragg peak enable greater healthy tissue sparing and offer patients a treatment option with fewer side effects when compared to traditional photon therapy [1–3]. Since its proposed use by Robert Wilson [4] in 1946, a total of 63 proton facilities are treating patients around the world [5]. Technologic advancements in proton therapy are continually being realized, thus increasing its availability as a treatment modality. There are currently 40 proton therapy facilities under construction, and 21 more in the planning stage in the world [5].

A common technique for proton beam delivery is passive scattering [6]. Using this technique, a narrow, nearly monoenergetic, proton beam is transformed to a clinically useful size by using scattering foils to laterally spread and flatten the beam and a range modulator wheel to increase the width of the Bragg peak [7–9]. The final components that the beam pass through are patient-specific devices, consisting of an aperture to collimate the field, and a range compensator to distally shape the beam [10–13]. A challenge that arises from use of the passive scattering proton beam delivery technique is the determination of beam output that is needed to deliver a prescribed dose, namely, calibrating the dose per monitor unit (DMU). Differences in range, modulation width, and beamline components for a treatment field will all affect the dosimetric output from a passive scattering treatment unit. Currently, there is a lack of a widely established procedure for output calculations [14]. Analytic, correction-based, and simplified Monte Carlo (MC) techniques have been proposed, but owing to the complexity and specificity of each individual treatment system, a global protocol has yet to be accepted [15–18]. Many proton facilities choose to use the calculation of DMU as a secondary check and rather perform measurement in a water equivalent phantom to calibrate the DMU to the patient [17].

A challenge associated with machine output prediction is characterizing the effects of the patient-specific compensator and patient anatomy on monitor unit (MU) calibration [19]. Analogous to photon therapy, implementation of these effects have been suggested as correction factors to the final calibrated DMU. The so-called compensator scatter factor (CSF) is responsible for taking into account compensator effects and is defined as the ratio of DMU under treatment conditions with and without the compensator in place in a homogenous phantom. In many cases, the CSF is ignored owing to the cumbersome nature of taking measurements for every patient's field-specific compensator. In fact, Fontenot et al [20] concluded that the high-dose gradients caused by the irregular shape of range compensators have the potential to make accurate calibration of DMU difficult, and they suggested that calibrations be done without consideration of the compensator. However, Akagi et al [21] reported that the effects of scatter from a compensator and patient anatomy could potentially result in a DMU difference of 2% to 3% from what is measured in a water phantom without the compensator.

The second factor that accounts for the effect of patient anatomy on DMU is the patient scatter factor (PSF). It is defined as the ratio of DMU in patient under treatment conditions to that in a homogenous medium [16]. During calibration, the effect of the PSF is neglected entirely owing to the limitations of measuring the effects of patient inhomogeneities on the DMU. Akagi et al [21] have demonstrated the possibility of using the pencil beam algorithm (PBA) in the treatment planning system (TPS) as a means of calculating the PSF. However, the study suggested that errors on the order of 2% to 3% are possible in the TPS-calculated PSF owing to limitations of the PBA [21]. In one recent study involving prostate cases, measurements in an anthropomorphic phantom showed that the PSF was negligible on the DMU [19]. However, a self-proclaimed limitation of this study was that the results were valid for only prostate cases, and to come to a more global conclusion, other sites needed to be investigated [19].

Monte Carlo (MC) simulation is widely accepted to be the most accurate technique for proton treatment dose calculation [22, 23]. Monte Carlo dose calculation is especially valuable in highly heterogeneous fields, where analytic techniques show limitations [24, 25]. Owing to the difficulties in estimation of the PSF and CSF through measurement, the most accurate way to calculate these factors and implement them in the DMU calibration is likely through MC simulation. However, there are technical drawbacks associated with this technique. Perhaps the most significant hurdle in this technique includes the long calculation times to run enough histories to gather significant data [26]. A previous study from Paganetti et al [25] indicated that calculation time could reach upwards of 6 hours to calculate dose in patient, which proved to be too inefficient for clinical use. Along with the extended calculation times, challenges also arise with MC in the complexities of modeling the proton treatment conditions accurately. For MC use to augment the treatment planning process with PSF and CSF calculations, accurate beamline and patient modeling are of great importance [27]. This requires a significant amount of technical skill by the user.

Currently, when the MUs of treatment fields are determined by using the measured dose from a verification plan in water without compensator, it is assumed that the TPS correctly calculates the CSF and the PSF in the patient plan, and thus the DMU in the patient. The purpose of this work is to show that this assumption may not be always valid, and the delivered dose to patient, based on the MU determined in water without compensator, may differ from the planned dose owing to the inaccuracies in the calculation of CSF and PSF by the proton PBA used in the TPS. For these factors to be included in output calculations, a deep investigation into the properties of the CSF and PSF is needed. To the best of the authors' knowledge, the current literature lacks detailed analysis on what causes a PSF or CSF to deviate from unity, and most conclusions drawn are limited to specific sites. With a thorough understanding of what influences the values of these factors, conclusions can be drawn that can apply to any treatment site, regardless of method of calculation used. The goal of this work is 2-fold: the first is to compare the PBA and MC calculation techniques for a wide range of treatment sites and fields, and the second is to investigate the properties that influence the PSF and CSF, and how to incorporate these factors in all treatment situations.

## Materials and Methods

### PSF and CSF Study

PSFs and CSFs for brain, lung, pancreas, and prostate treatment sites were calculated by using MC simulation and PBA from the TPS software to evaluate the agreement between the two.

### PSF and CSF Using the PBA

**Figure 1:**
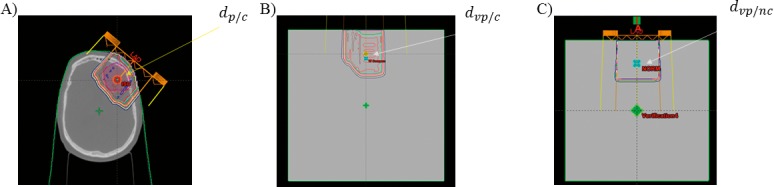
An example of the PSF and CSF calculation points using the treatment planning system verification plan tool for a brain case at a WED of 5 cm. (A) Calculation point in the patient. (B) Calculation in the phantom via the verification plan tool with the compensator in place. (C) The same calculation as (B) without the compensator. Abbreviations: CSF, compensator scatter factor; PSF, patient scatter factor; WED, water equivalent depth.

To calculate the PSF and CSF by using the PBA in the TPS, the same method was followed as described by Sahoo et al [16]. Thirty-eight treatment fields (8 brain, 8 lung, 11 pancreas, and 11 prostate) were taken from anonymized patient treatment plans of patients who consented to participating in institutional review board-approved research. The dose was calculated at isocenter, which was located in the center of the PTV along the central beam axis, in each field. A corresponding plan was then created by using the “verification plan” tool in the TPS. The proton fluence, aperture, and compensator were copied from the treatment field and dose was calculated at the same water equivalent depth (WED) along the central beam axis in a homogenous water phantom. The value of the PSF was then acquired for each field, using Equation 1. The compensator was then removed from the verification plan, and the dose at the same equivalent WED was recalculated. The value of CSF was then acquired using Equation 2.

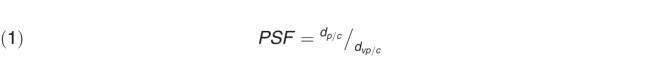


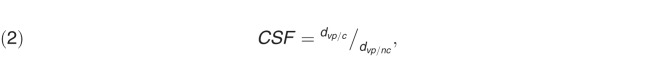
where *d_p_*_/_*_c_* is the dose in patient, *d_vp_*_/_*_c_* is the dose in the verification plan at the same WED, and *d_vp_*_/_*_nc_* is the dose at the same WED in the verification plan without the compensator in place. The workflow has been shown in **Figure 1** for a typical brain case.

### PSF and CSF Calculated in MC

Three fields from each site used to calculate the PSF and CSF factors in the TPS were also used to calculate the same factors in MC simulation. The TOPAS (TOolkit for PArticle Simulation) version 3.1 [28] simulation code was installed on our T630 server, using 2 Intel Xeon E5 2660 V4 2 GHz processors, and used for all of the MC calculations. TOPAS has been shown to be a prominent and user-friendly simulation code for proton therapy [29]. Phase space files from the recently modeled beamline for our Mevion S250 double-scattering proton therapy system was used as the source for simulation of the patient treatment plans [30]. The treatment parameters for each field were exported from the TPS into the simulation space, along with the patient CT images. HU values from each pixel were then converted to corresponding materials and density by using the technique proposed by Schneider et al [31]. Patient-specific apertures and compensators were exported from the TPS and converted to STL files for implementation in the MC space.

For all simulations, the TOPAS default physics package was used, which includes “g4em-standard_opt4,” “g4h-phy_QGSP_BIC_HP,” “g4decay,” g4ion-binarycascade,” “g4h-elastic_HP,” and “g4stopping.” The range cut used in simulation was 0.05 mm, corresponding to an energy cut in water of 990 ev, 57.3 keV, 5 keV, and 56.6 keV for gamma rays, electrons, protons, and positrons, respectively. All dose quantities were scored as dose to medium, given in mGy. For each field, 50 million histories were used to reduce the statistical uncertainty to a negligible level.

### Phantom Study

A benchmark study was carried out to ensure the integrity of our simulation technique in an anthropomorphic phantom. A 2-field treatment plan was first created for a brain case in a RANDO phantom. The brain case was chosen owing to the simplicity of the site, as well as the wide range of HU values that would be present in the CT data set. For the physical measurement, 100 MUs were delivered to the RANDO phantom under treatment conditions and a dose measurement at isocenter was taken with a thermoluminescent detector (TLD). Two more TLD measurements were taken under the same treatment parameters with a water equivalent phantom substituted in for the RANDO phantom. One measurement was taken with the compensator in place, and the other without. The dose measurements taken from the TLD were used in Equations 1 and 2 to calculate the measured PSF and CSF. The same treatment fields were then simulated in MC and also calculated by using the PBA in the TPS for comparison purposes.

### Evaluation of PSF

The main function of the PSF is to account for patient anatomy on DMU. Therefore, to investigate how different tissue types influence the PSF, a sample of the prostate and lung treatment fields were broken down into groups of materials, based on the HU values within the field. The prostate cases were chosen to isolate how bone influences the PSF, whereas the lung cases were chosen to investigate the influence of air. Using the TPS, the 50% isodose curve was contoured to a structure, and a histogram of HU values from the created structure was produced for each field. Five different material types were grouped and assigned their percentage contribution in the field according to their HU, namely, air (−1000 to −650 HU), thin tissue (−651 to −500 HU), soft tissue (−501 to 125 HU), bone (126 to 500 HU), and dense bone (501 to 1245 HU). The percentage of materials present was then compared to the calculated PSF values for the field.

### Evaluation of CSF

From the 38 CSF factors calculated, we chose 7 that best reflected the range of values attained and calculated the interquartile range (IQR) of thickness values of the compensator around the central beam axis. The IQR was chosen as a metric of irregularity of compensator shape, owing to its ease of calculation, and its stability in the presence of extreme values in the data set. The area around the central beam axis chosen to calculate the IQR was determined through calculation of the maximum scattering distance of the beam with respect to beam energy by the General Highland approximation [32].

## Results and Discussion

### PBA and MC Comparison

The results from the benchmark case in the Rando phantom showed good agreement between MC simulation and measurement. The measured PSF was 1.045 and the MC-calculated PSF was 1.031, giving a difference of only 1.4%. The difference between the measured and MC-calculated CSF was only 0.7%, with the measured and MC factors being 1.023 and 1.016, respectively. This agreement suggested that our MC model was adequate for further PSF and CSF calculations and was a good point of comparison for the PBA calculation method.

**Table 1** shows the PBA- and MC-calculated PSF and CSF factors for the 12 fields compared. The MC- and PBA-calculated PSF factors agreed to within 4% of each other. The average differences between MC and PBA factors for the brain, pancreas, prostate, and lung sites were 1.6%, 1.0%, 0.6%, and 1.9% respectively. For each field, both prediction techniques were in agreement as to when the factors were greater than or less than unity. In nearly all cases, the MC-calculated PSFs were larger in magnitude than the PBA-calculated PSFs. This suggests that our MC model predicts the scatter from surrounding patient anatomy to play a more prominent role in the DMU than does the PBA. The limitations of the PBA in TPS to accurately account for proton scatter in heterogeneous media has been well documented before this study, [24, 32] and it is believed that this resulted in the discrepancies between PBA- and MC-calculated PSF factors. This is clearly shown in the lung cases, where there was the most inhomogeneity present in the patient anatomy, and disagreement between the MC and PBA was the largest.

**Table 1:** A comparison of the CSF and PSF factors calculated in both MC and the PBA for 4 different target locations: brain, pancreas, prostate, and lung (3 fields for each site).

**Locations**	**Fields**	**TPS CSF**	**MC CSF**	**TPS PSF**	**MC PSF**
Brain	1	1.010	1.024	1.003	1.030
	2	1.002	1.000	1.002	1.000
	3	1.004	1.000	1.005	1.028
Pancreas	1	1.000	1.002	1.002	1.005
	2	1.033	1.030	1.002	1.014
	3	1.011	1.017	1.002	1.017
Prostate	1	1.016	1.006	1.023	1.025
	2	1.010	1.010	1.013	1.025
	3	1.003	1.002	1.017	1.022
Lung	1	1.012	1.026	0.961	0.960
	2	1.009	1.014	0.920	0.947
	3	1.020	1.017	0.919	0.951

**Abbreviations:** CSF, compensator scatter factor; PSF, patient scatter factor; MC, Monte Carlo; PBA, pencil beam dose algorithm; TPS, treatment planning system.

The CSFs calculated by using both techniques agreed very well. The largest discrepancy between MC and PBA factors was 1.4%, with the average differences for each site being at or below half a percent. These data suggested that the PBA was adequate for predictions on the impact of the compensator on the output calculation. The improved agreement of the CSF between the 2 methods of calculation when compared to the PSF was most likely because the PBA needed only to consider scatter from Lucite/air interfaces for the CSF factors. The PSF was much more complex in that many different types of material interfaces existed with a wide range of atomic makeup and densities. Owing to the limitations of the PBA to model scatter, it was reasonable to see larger discrepancies in the PSF calculation methods than for the CSF.

### PSF and CSF PBA Results

**Table 2** shows the remaining 26 CSF and PSF factors calculated by using the PBA in the TPS for each of the brain, pancreas, prostate, and lung treatment plans studied.

**Table 2:** The PBA-calculated CSF and PSF factors for each patient plan.

**Patient**	**Location**	**CSF**	**PSF**	**CPSF**
A	Brain	1.018	1.010	1.028
B	Brain	1.001	1.002	1.003
C	Brain	1.000	1.004	1.004
D	Brain	1.006	0.993	0.999
E	Brain	1.006	0.986	0.992
F (field1)	Pancreas	1.010	0.998	1.008
F (field2)	Pancreas	1.018	1.005	1.023
G	Pancreas	1.014	0.993	1.007
H	Pancreas	1.005	1.000	1.005
I (field1)	Pancreas	1.009	1.004	1.013
I (field2)	Pancreas	1.025	0.981	1.006
J (field1)	Pancreas	1.020	1.020	1.040
J (field2)	Pancreas	1.019	1.019	1.038
K (field1)	Prostate	1.010	1.013	1.023
K (field2)	Prostate	1.014	1.010	1.024
L (field1)	Prostate	1.028	1.004	1.032
L (field2)	Prostate	1.038	1.005	1.043
M (field1)	Prostate	1.006	1.014	1.020
M (field2)	Prostate	1.006	1.008	1.014
N (field1)	Prostate	1.002	1.002	1.004
N (field2)	Prostate	1.017	1.008	1.025
O	Lung	1.044	0.971	1.014
P	Lung	1.015	0.977	0.992
Q	Lung	1.008	1.000	1.008
R	Lung	1.020	0.947	0.966
S	Lung	1.007	0.944	0.951

Abbreviations: PBA, pencil beam dose algorithm; CSF, compensator scatter factor; PSF, patient scatter factor; CPSF, Compensator scatter factor times patient scatter factor.

### PSF Evaluation

**Figure 2:**
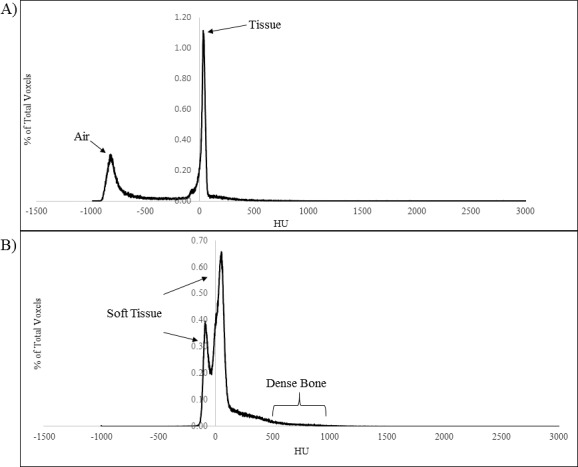
(A) A histogram of CT values in the treatment field for patient S and (B) a histogram of CT values in the treatment field for patient K. Abbreviations: CT, Computed Tomography; HU, Hounsfield Unit

The value of the PSF appeared to be heavily dependent upon material in the beam's eye view (BEV). Sites with large quantities of air in the BEV (such as lung cases) yielded a PSF less than 1. Air, having much less scattering power than water, resulted in less scatter contribution to the point of measurement than that in phantom. **Figure 2A** shows a histogram of CT values for patient S, where there are large amounts of air present.

**Figure 3:**
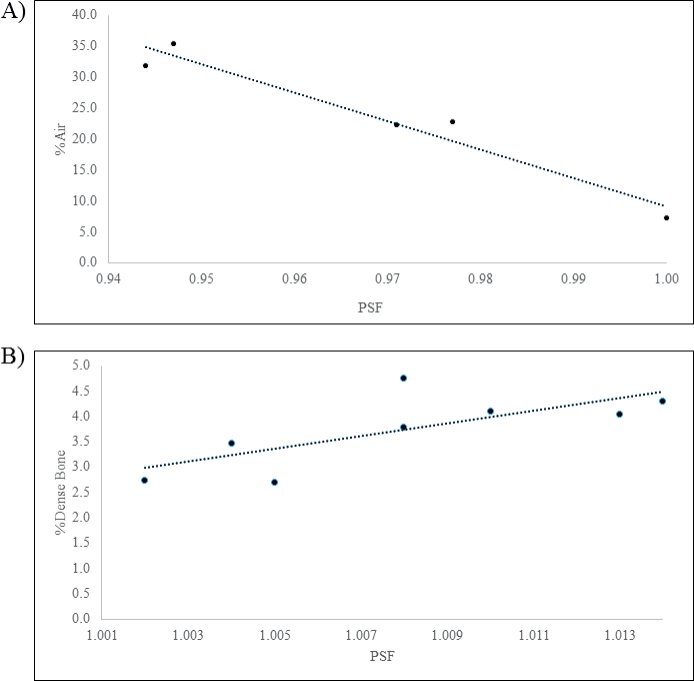
(A) The relationship between the PBA-calculated PSF and the percentage of air in the treatment field for the lung cases. (B) The relationship between the PBA-calculated PSF and the percentage of dense bone in the treatment field for the prostate cases. Abbreviations: PBA, pencil beam dose algorithm; PSF, patient scatter factor.

The first peak shown at −800 HU was representative of the quantity of air in the field, while the second peak around 0 HU was soft tissue. The area under these 2 peaks gave a quantitative breakdown of the percentage of each material present. For patient S shown, the field contained 32% air, 62% soft tissue, and 6% bone, yielding a PSF of 0.944. According to the data, as the quantity of air present in the BEV of the field increased, the corresponding PSF decreased (**Figure 3A**). These data suggested that the PSF for lung cases was important to account for, having values that could be nearly 6% lower than unity. The wide range of values could be attributed to the many different locations in which a target might be present. Situations where the target was located at or near the chest wall had less air present in the beam path, resulting in a PSF closer to unity. However, the inverse situation where the target was located toward the midline of the chest, where the beam must traverse greater amounts of air, resulted in a large deviation of the PSF from unity.

Fitting a linear function to the results yielded Equation 3.

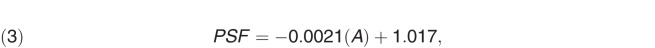
where *A* is the percentage of air in the field. Owing to the wide range of scenarios present in lung treatments, this equation could be used as a cursory guideline to estimate PSF from the amount of air present in the treatment field.

Conversely, sites such as the prostate that have a significant amount of dense bone (HU > 500) in the BEV produced a PSF greater than 1. This was because dense bone had more scattering power on the incoming fluence of protons, resulting in more scatter delivering dose to the point of interest than in a homogenous water phantom. A histogram of HU values for field 1 of patient K is shown in **Figure 2B**.

The first 2 peaks in the histogram are representative of the soft tissue present in the BEV, with the first peak representing the prostate gland. The shoulder of the second peak beginning at about 125 HU is where the soft tissue values turn to bone, and the tail of the shoulder starting at 500 HU represents the dense bone. The material breakdown for the particular case of field 1 of patient K is 0% air, 80.8% soft tissue, 15.1% bone, and 4.1% dense bone. The data range of values for sites with bony anatomy present was narrow. For the prostate cases, the spread of values of dense bone present was 2.1%, resulting in only a 1.2% range of PSF values. The largest value of PSF for all sites studied was just 2.0% greater than unity. This low variability in the data could create difficulties in analyzing how strongly the effects of dense bone were on the PSF. However, according to **Figure 3B**, there does appear to be a correlation between the amount of dense bone present in the treatment field and the PSF.

Fitting a curve to the data yielded Equation 4, which could be used as a preliminary estimate to predict the magnitude of the PSF, based upon the amount of dense bone in the field.

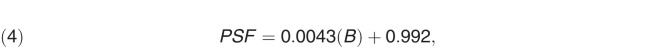
where *B* is the percentage of dense bone in the field.

The data clearly showed that the prostate cases studied always resulted in a PSF greater than 1 owing to the bone in the field, while the opposite was true for lung cases, where there were large amounts of air present. However, similar conclusions could not be drawn for the other treatment sites examined in this study. Both the brain and pancreas cases could result in a PSF greater than 1 or less than 1. This was because these sites could have bony anatomy and air present in the treatment field, depending on the beam angles and locations of the targets. For example, a pancreas site might have bone in the field from a rib or air in the field from the bowel. This presented 2 competing factors on the PSF, which was difficult to predict when using analytic methods. The same was true for brain cases where bone was present from the skull, but there might also be air in the field from a sinus. In some special circumstances, a gap between the contoured immobilization device and the patient surface could introduce an air cavity, which could also influence the PSF. Sites that have multiple types of inhomogeneities will need to be evaluated on a case-by-case basis to ensure that the proper PSF be assigned.

### CSF Evaluation

**Figure 4:**
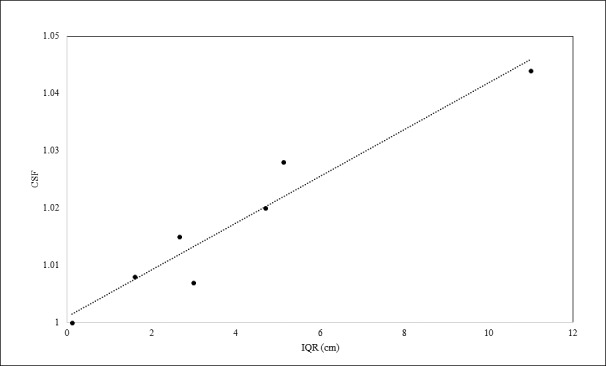
The relationship between the IQR of the compensator thickness and the calculated CSF. Abbreviations: CSF, compensator scatter factor; IQR, interquartile range.

A general observation of the CSF was that all calculated factors were equal to or greater than 1. The range of values extended from 1.000 to 1.044. This suggested that the presence of the compensator tended to increase the amount of scatter to the point of measurement, thus adding dose. The wide range in CSF factors could be attributed to the degree of irregularity of compensator shape. **Figure 4** shows the CSF factors as a function of IQR of compensator thicknesses around the field area that could contribute scatter to the calibration point for 7 cases.

There was a clear linear relationship between the IQR and the CSF. A large IQR suggested a wide range of compensator thicknesses in the region of interest, which was a direct indication of a nonuniform compensator shape. Large thickness irregularities on the periphery of the region of interest contributed more scatter fluence to the point of measurement than fluence that was scattered out of the central beam axis. Because compensator thickness at any point was determined by the WED along a ray from the source to the distal end of the target [33], it is concluded that the distribution of patient anatomy around the point of measurement will influence the compensator shape. Treatment fields with minimal tissue inhomogeneities present in the beam's eye view will result in a more uniform range compensator, which in turn will result in a CSF closer to unity. Based on the current data, a functional form shown in Equation 5 can be used to predict the CSF, based on the IQR.

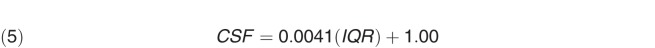



Using Equation 5, the cumbersome procedure of calculating the CSF using the MC or PBA technique is eliminated.

## Conclusions

Based on the data collected, it is validated that the compensator and patient anatomy can have a significant impact on the DMU. The CSF in some cases is nearly 4% above unity, while values of the PSF can be as low as 8% below unity in lung cases, or as large as 2% greater than unity in some of the pancreas cases. The wide range of values for the PSF suggest inclusion is critically important, especially in cases where there are large tissue inhomogenities such as the lung or pancreas. It is recommended that the PSF and CSF should always be taken into account together. This is especially true in situations where the PSF is less than 1, because the two factors can have a canceling effect. In those cases, only including one of the scatter factors in the DMU calculation could result in a large error.

In clinical practice, the excellent agreement between the MC and PBA-calculated CSF factors suggests that the TPS can be used to routinely calculate the CSF for inclusion in the DMU. As a secondary check, one could also estimate the factor from the IQR by using Equation 5 shown previously. The method of attaining the PSF is slightly more challenging. It is well documented that the PBA has limitations in dose calculation for heterogeneous media, and there are slight discrepancies between our simulation model and what the TPS calculated. The largest discrepancies between the MC and TPS are in cases where the heterogeneities in the treatment field are the largest. This is clearly shown in the lung sites, where the average differences are nearly 2%. Therefore, it is recommended that the treatment fields be optimized to reduce the magnitude of heterogeneity within the treatment field. This strategy will have a 2-fold effect on the PSF, the first being that reducing the amount of inhomogeneity (such as bone and air) in the field will keep the PSF close to unity, as shown in this study. This in turn minimizes the influence of patient anatomy on the output calculation. The second benefit of this strategy is that a more homogenous treatment field tends to show better agreement between MC and PBA PSF calculation methods, suggesting that use of the TPS for PSF calculation is a viable option. In a clinical situation where the TPS-calculated PSF deviates from unity by greater than 3%, MC should be used as a secondary check to investigate the PSF further. One may also choose to consult Equations 3 and 4 as a third check. However, a limitation of Equations 3 and 4 is that they apply only in situations where there is strictly air or bone in the field, but not when there is an appreciable amount of both.

A limitation of this work stems from the location of the point of interest where the CSF and PSF factors were calculated. In this study, the point was on the central beam axis in the middle of the SOBP. However, in some situations this point was located in a dose gradient region, and it was observed that small shifts (1-2 mm) of the point to reach a lower gradient region could result in large changes in the scatter factor calculation, on the order greater than a percent. Because the point of DMU calibration does not always coincide with the middle of the SOBP along the central beam axis, future studies should examine the effect of the location of the point of calculation for the CSF and PSF calculations.

Overall, this study has provided a better understanding of the influences of the compensator and patient anatomy on the DMU. We also believe that it can help to formulate a strategy for inclusion of these factors in the final output calculation, which we deem necessary for the most accurate delivery of patient dose.
